# The Zinc Transporter SLC39A14/ZIP14 Controls G-Protein Coupled Receptor-Mediated Signaling Required for Systemic Growth

**DOI:** 10.1371/journal.pone.0018059

**Published:** 2011-03-22

**Authors:** Shintaro Hojyo, Toshiyuki Fukada, Shinji Shimoda, Wakana Ohashi, Bum-Ho Bin, Haruhiko Koseki, Toshio Hirano

**Affiliations:** 1 Laboratory for Cytokine Signaling, RIKEN Research Center for Allergy and Immunology, Suehiro, Tsurumi, Yokohama, Kanagawa, Japan; 2 Department of Allergy and Immunology, Osaka University Graduate School of Medicine, Osaka University, Yamada-oka, Suita, Osaka, Japan; 3 Department of Anatomy-1, Tsurumi University School of Dental Medicine, Tsurumi, Yokohama, Kanagawa, Japan; 4 Laboratory for Developmental Genetics, RIKEN Research Center for Allergy and Immunology, Suehiro, Tsurumi, Yokohama, Kanagawa, Japan; 5 Laboratory of Developmental Immunology, JST-CREST, Graduate School of Frontier Biosciences, Graduate School of Medicine, and WPI Immunology Frontier Research Center, Osaka University, Yamada-oka, Suita, Osaka, Japan; Universidade Federal do Rio de Janeiro, Brazil

## Abstract

Aberrant zinc (Zn) homeostasis is associated with abnormal control of mammalian growth, although the molecular mechanisms of Zn's roles in regulating systemic growth remain to be clarified. Here we report that the cell membrane-localized Zn transporter SLC39A14 controls G-protein coupled receptor (GPCR)-mediated signaling. Mice lacking *Slc39a14* (*Slc39a14*-KO mice) exhibit growth retardation and impaired gluconeogenesis, which are attributable to disrupted GPCR signaling in the growth plate, pituitary gland, and liver. The decreased signaling is a consequence of the reduced basal level of cyclic adenosine monophosphate (cAMP) caused by increased phosphodiesterase (PDE) activity in *Slc39a14*-KO cells. We conclude that SLC39A14 facilitates GPCR-mediated cAMP-CREB signaling by suppressing the basal PDE activity, and that this is one mechanism for Zn's involvement in systemic growth processes. Our data highlight SLC39A14 as an important novel player in GPCR-mediated signaling. In addition, the *Slc39a14*-KO mice may be useful for studying the GPCR-associated regulation of mammalian systemic growth.

## Introduction

Zn is an essential trace element that is involved in diverse cellular events by affecting the structural and catalytic functions of enzymes and transcription factors [1], [2]. Zn homeostasis is tightly controlled by two types of Zn transporters, SLC39/ZIP importers and SLC30/ZnT exporters [3], [4], and by metallothioneins (MTs) [5], all of which participate in various physiological events. For instance, SLC39A4/ZIP4 is expressed in the intestine and has a role in Zn uptake through enterocytes [6], [7]. Patients with Acrodermatitis Enteropathica (AE), who suffer from severe skin disease and frequent infections, have unusually low serum concentrations of Zn due to pathogenic loss-of-function mutations in SLC39A4 [6], [7]. Another Zn transporter, SLC39A13/ZIP13, is expressed on the Golgi and controls the intracellular Zn distribution in cells of mesenchymal origin. *Slc39a13*-KO mice show dwarfism and abnormal bone and cutaneous development, and a loss-of-function mutation in this gene was found in patients with a novel type of Ehlers-Danlos Syndrome (EDS), demonstrating that SLC39A13 plays a critical role in growth control and connective tissue formation in mouse and human [8]. In fact, considerable evidence indicates that Zn transporters are involved in regulating a variety of intracellular signaling pathways in animals, from flies to vertebrates [8], [9], [10], [11], [12], [13]. In addition, Zn is reported to act as a neurotransmitter [14] or rather, as an allosteric regulator for a Zn-sensing receptor [15], and as an intracellular signaling molecule [11], [16], [17].

Somatic growth, which affects body size, is regulated by endogenous and systemic factors, such as nutrients, hormones, and growth factors [18], [19]. The production of growth hormone (GH) in the pituitary gland and of insulin-like growth factor (IGF-I) in the liver are the main endocrine influences on somatic growth [20]. GH and IGF-I regulate longitudinal bone growth by controlling endochondral ossification, a defined sequence of events underlying chondrocyte differentiation in the growth plate [18],[21],[22].

Aberrant Zn homeostasis is associated with vertebrate growth retardation [1], [8], [20] and metabolic disorders [4], [23]. In particular, Zn deficiency causes dwarfism with reductions in the circulating GH and IGF-I concentrations [20], and decreased growth-plate width, which is correlated with reduced cellular Zn content [24]. Intriguingly, the growth retardation cannot be reversed by maintaining circulating levels of GH or IGF-I through exogenous administration in Zn-deficient animals [20]. These findings collectively suggest that Zn's uptake into cells and the subsequent intracellular Zn accumulation affect the hormone signaling cascade(s) required for GH production and chondrocyte differentiation. Since the endocrine system consists of a complex group of glands involved in growth and metabolism, its perturbation can broadly affect human health, and it is important to elucidate the mechanisms underlying the early stages of endocrine disorders. However, the molecules responsible for Zn homeostasis have been elusive, and how Zn affects the intracellular signaling that regulates growth-related endocrine processes has been little studied.

SLC39A14, a SLC39/ZIP family member, transports the Zn ion into cells in an *in vitro* culture system [25], [26], but its physiological roles are still speculative. Here, we show that SLC39A14 plays important roles in mammalian growth and energy metabolism by regulating GPCR-cAMP-CREB signaling. Our findings unexpectedly revealed the biological contribution of a Zn transporter to GPCR-mediated signaling, which may help to explain why Zn deficiency results in growth inhibition and metabolic and endocrine-related disorders.

## Results

### *Slc39a14*-KO mice show dwarfism and shortened long bones

Since Zn-deficient animals with growth retardation show reduced amounts of cellular Zn [20], it has been assumed that Zn uptake and its subsequent intracellular accumulation in pituitary cells and chondrocytes, which are respectively important for GH production [27] and bone elongation [21], might be a common, critical initial step required for growth regulation. Therefore, first, to identify SLC39 members that might contribute to the influx of Zn into pituitary cells and chondrocytes, we examined the mRNA expression levels of SLC39 Zn transporters in these cells. Among the SLC39 family members, SLC39A14 was expressed in both types of cells (**Figure S1**). In addition, *Slc39a7* was abundant, and *Slc39a1*, *6*, and *13* showed relatively high expression levels in both cell types. SLC39A7 and SLC39A13 are present mainly in intracellular organelles [8], [28], [29], and SLC39A1 and SLC39A6 are localized to both the plasma membrane and intracellular organelles [30], [31], [32], [33]. *Slc39a1*-deficient mice are reported to display no obvious phenotypes under normal growth conditions [34]. *Slc39a8* and *Slc39a10* were highly expressed in chondrocytes but poorly in pituitary cells. Since SLC39A14 is so far reported to be a SLC39 member that resides only on the plasma membrane [25], [26], [35], we chose to examine SLC39A14 further as a candidate regulator of systemic growth via Zn influx.

**Figure 1:**
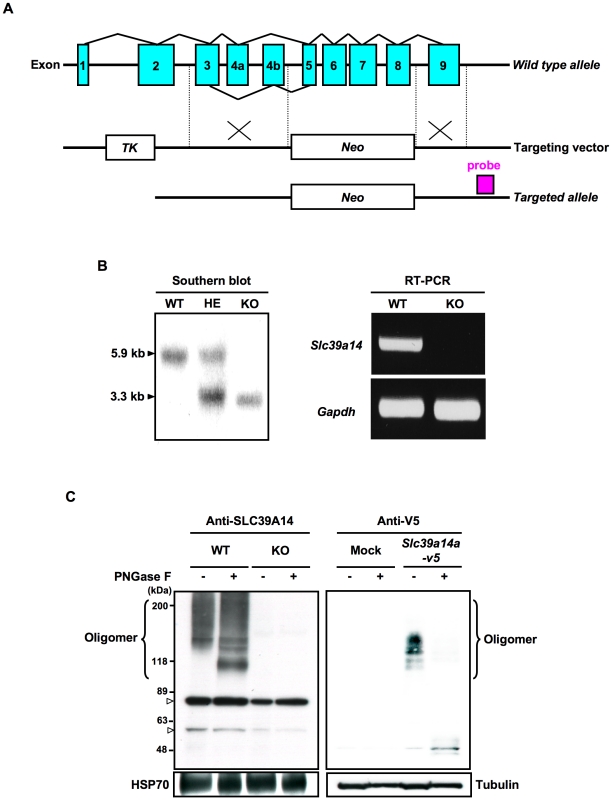
Generation of *Slc39a14*-KO mice. (**A**) Schematic diagram of the construct used to generate *Slc39a14*-KO mice. The *Slc39a14* genomic structure shows the alternatively spliced exon 4. Exon 4a encodes SLC39A14A, and exon 4b encodes SLC39A14B. (**B and C**) Homologous recombination by crossing heterozygotes was confirmed by southern blotting analysis (B, left) using genomic DNA from the tail, RT-PCR (B, right) using the liver mRNA, and western blotting analysis using liver whole-cell lysate (C, left), from litter mates. Cell lysates from wild-type, *Slc39a14*-KO liver (C, left), or *Slc39a14*a-v5 293T cells (C, right) were treated with or without PNGase F, and then subjected to SDS-PAGE and immunoblotting with antibodies to SLC39A14 or V5. The oligomeric bands are indicated. Non-specific bands.

To investigate the physiological role of SLC39A14 in growth, we generated mice with a deletion in the *Slc39a14* gene. We constructed a targeting vector designed to eliminate the genomic region encompassing exons 5–8, which contain the histidine-rich domain and conserved HEXPHEXGD motif that is common to SLC39 family members [36], and to delete both alternative splice variants of SLC39A14, designated SLC39A14A and SLC39A14B, encoded by exons 4a and 4b, respectively [35] (**Figure 1A**). After electroporation of this vector into embryonic stem (ES) cells, the correctly targeted ES cell clones were identified by southern blotting analysis (**Data not shown**). Homologous recombinant ES cell clones were used to generate *Slc39a14*-KO heterozygotes, which were intercrossed to yield *Slc39a14*-KO homozygotes. Southern blotting analysis of the genomic DNA of wild-type (WT), heterozygous (HE), and homozygous (HO) mice confirmed the deletion of *Slc39a14* in the *Slc39a14*-KO mice (**Figure 1B**). The gene knockout was further confirmed by the lack of its mRNA (**Figure 1B**). SLC39A14 protein is posttranslationally modified [25], [26]. Western blotting analysis with anti-mouse SLC39A14 and anti-V5 antibodies demonstrated that a group of high-molecular-mass bands (compatible with glycosylated oligomers; >89 kDa) of endogenous SLC39A14 were abundant in the cell lysates of both wild-type liver and *Slc39a14a-v5*-overexpressing 293T cells, whereas these bands were completely absent from lysates of *Slc39a14*-KO liver (**Figure 1C**). These data suggested that SLC39A14 was successfully deleted in the *Slc39a14*-KO cells.

**Figure 2:**
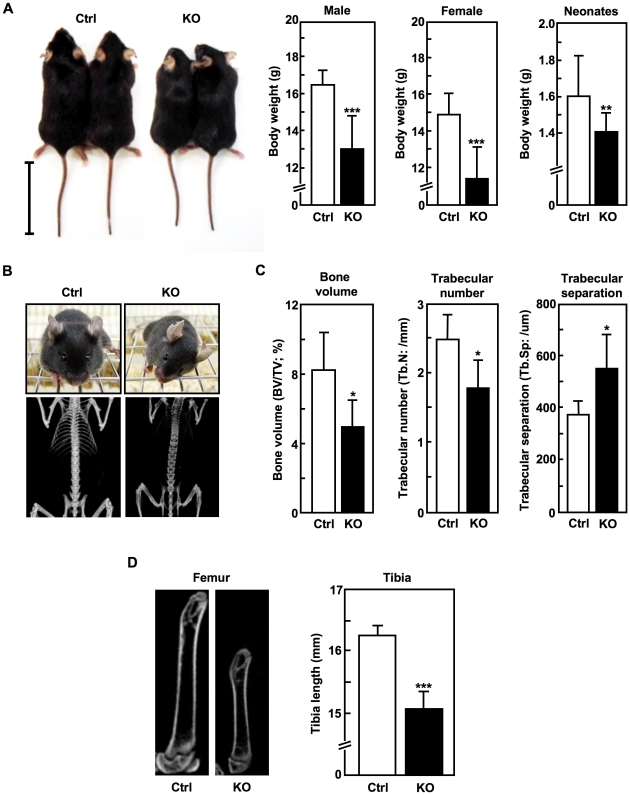
Dwarfism, torticollis, scoliosis, osteopenia, and shortened long bones in *Slc39a14*-KO mice. (**A**) **Left.** Appearance of control (Ctrl) and *Slc39a14*-KO mice (4-weeks-old). Bar indicates 5 cm. **Right.** Body weights of 4-week-old control (Ctrl) (male, n = 9; female, n = 9), *Slc39a14*-KO (male, n = 10; female, n = 7), and neonatal (control, n = 22; *Slc39a14*-KO, n = 14) mice. Data represent the mean ± S.D. (**P<0.01, ***P<0.001). (**B**) Frontal views (4-week-old) and X-ray radiographs (8-week-old) of a control (Ctrl) and a *Slc39a14*-KO female mouse. (**C**) Bone histomorphometric analysis of 4-week-old control (Ctrl) and *Slc39a14*-KO female mice (n = 5). Data represent the mean ± S.D. (*P<0.05). (**D**) X-ray radiographs of the femurs (8-week-old), and the tibial length (6-week-old) of control (Ctrl) and *Slc39a14*-KO mice (n = 5). Data represent the mean ± S.D. (***P<0.001).

The *Slc39a14*-KO mice exhibited growth retardation compared to control mice, and dwarfism was visible even in neonates (**Figure 2A**). The mice also exhibited torticollis (wry neck, **Figure 2B** **, upper**) and slightly radiolucent bones (**Figure 2B** **, lower**). Bone histomorphometric analysis showed that these moderate osteoporotic phenotypes were associated with decreased bone volume and trabecular number, and increased trabecular separation (**Figure 2C**). The length of the long bones in the *Slc39a14*-KO mice was also significantly reduced (**Figure 2D**), indicating that SLC39A14 may play critical roles in skeletal formation.

### Abnormal chondrocyte differentiation in the growth plate of *Slc39a14*-KO mice

**Figure 3:**
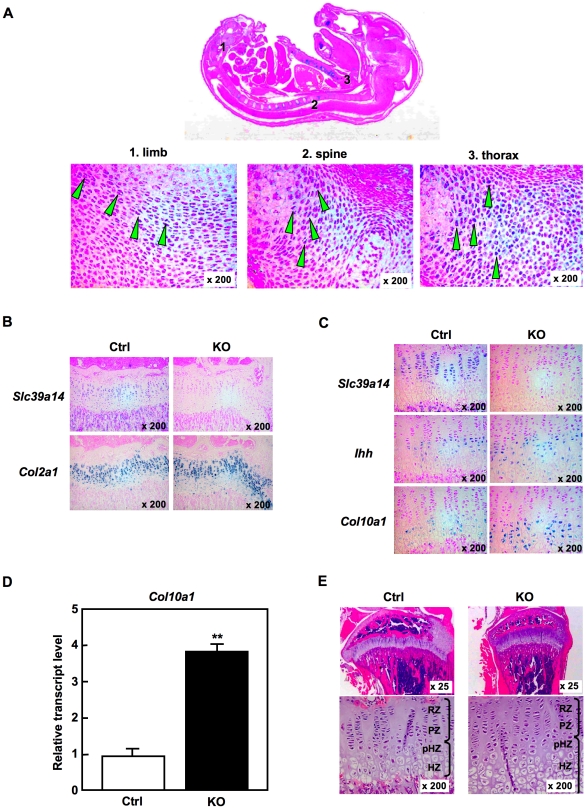
Abnormal chondrocyte differentiation in the growth plate of *Slc39a14*-KO mice. (**A**) *In situ* hybridization analysis for *Slc39a14* in an embryo at 16.5 dpc, and magnified images of the limb (1), spine (2), and thorax (3). Green arrowheads indicate *Slc39a14* expression. (**B and C**) *In situ* hybridization analysis for *Slc39a14*, *Col2a1, Ihh*, and *Col10a1* in the growth plates from 4-week-old control (Ctrl) and *Slc39a14*-KO mice. (**D**) *Col10a1* levels in control (Ctrl) and *Slc39a14*-KO chondrocytes. Data represent the mean ± S.D. (**P<0.01). (**E**) H&E staining of the growth plates from control (Ctrl) and *Slc39a14*-KO mice (4-week-old).

To investigate the role of SLC39A14 in bone, we first examined the expression profile of *Slc39a14* mRNA by *in situ* hybridization analysis. We found relatively high *Slc39a14* mRNA expression in bone tissues such as the limb, spine, and thorax of the E16.5 embryo (**Figure 3A**), leading us to examine the bone morphology of the *Slc39a14*-KO mice.

Endochondral ossification is a process required for the proper development of long bones and vertebrate growth [37]. In this process, resting chondrocytes differentiate into proliferative chondrocytes with a columnar morphology, stop dividing, and mature into prehypertrophic and then hypertrophic chondrocytes. We first asked whether the shorter long bones of the *Slc39a14*-KO mice resulted from a defect in this process. In the control growth plate, *Slc39a14* was expressed from the resting zone (RZ) to the prehypertrophic zone (pHZ), with relatively high abundance in the proliferative zone (PZ) and little in the hypertrophic zone (HZ) (**Figure 3B and C** **, top panels**). The *type II Collagen* (*Col2a1*) gene is exclusively expressed in the RZ and PZ in the growth plate, and its expression level was increased in the growth plate of the *Slc39a14*-KO mice compared to control mice (**Figure 3B** **, lower panels**). In addition, the expression of pHZ markers *indian hedgehog* (*Ihh*) and *parathyroid hormone 1 receptor* (*Pth1r*), were enhanced in the *Slc39a14*-KO growth plate (**Figure 3C** **, middle panels and Figure S2**), and *type X Collagen* (*Col10a1*), an HZ marker, was also upregulated in the growth plate and in cultured chondrocytes from the *Slc39a14*-KO mice (**Figure 3C** **bottom panels, and** **Figure 3D**). In line with these observations, both the pHZ and HZ were elongated, and the RZ and PZ were narrowed, indicating that hypertrophy was accelerated in the *Slc39a14*-KO growth plate (**Figure 3E**).

### SLC39A14 positively regulates PTH1R-cAMP-CREB signaling in chondrocytes

**Figure 4:**
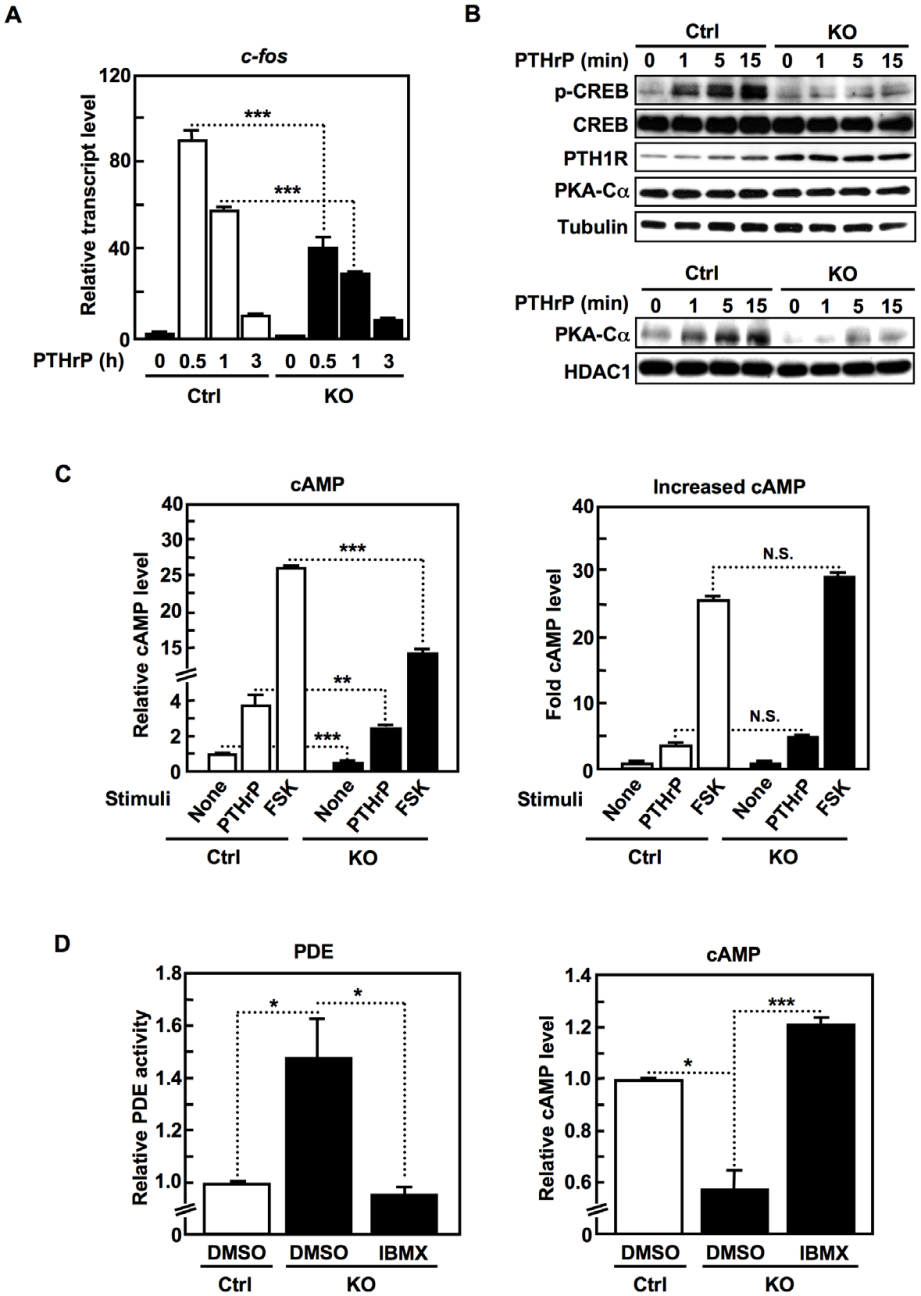
SLC39A14 positively regulates PTH1R-cAMP-CREB signaling in chondrocytes. (**A**) PTHrP-induced *c-fos* levels in control (Ctrl) and *Slc39a14*-KO chondrocytes. Data represent the mean ± S.D. (***P<0.001). (**B**) CREB phosphorylation and nuclear PKA-Cα translocation in control (Ctrl) and *Slc39a14*-KO chondrocytes with PTHrP treatment. Whole-cell lysate (upper panels) or the nuclear fraction (lower panels) was subjected to SDS-PAGE and immunoblotting with antibodies to the indicated proteins. (**C**) cAMP level and the fold-increase in cAMP level in control (Ctrl) and *Slc39a14*-KO chondrocytes before and after PTHrP or FSK treatment. Data represent the mean ± S.D. (**P<0.01; ***P<0.001; N.S., no significance). (**D**) Effect of IBMX treatment on the PDE activity and cAMP level in control (Ctrl) and *Slc39a14*-KO chondrocytes. Data represent the mean ± S.D. (*P<0.05, ***P<0.001).

A number of signaling pathways regulate the steps of chondrocyte differentiation in the growth plate [18], [21]. Among them, we were particularly interested in PTH1R signaling, because it regulates chondrocyte differentiation by suppressing hypertrophy in the growth plate [37], and the morphology of the *Slc39a14*-KO growth plate partly phenocopies that of PTH1R-mutant or -deficient mice [38], [39]. Parathyroid hormone-related peptide (PTHrP) stimulates the phosphorylation of cAMP response element-binding protein (CREB) by the nuclear-translocated catalytic alpha subunit of protein kinase A (PKA-Cα), and the phosphorylated CREB (p-CREB) then induces the transcription of *c-fos* [40]. As shown in **Figure 4A**, the PTHrP-mediated *c-fos* transcription was significantly reduced in the *Slc39a14-*KO chondrocytes. The induction of CREB phosphorylation and the nuclear translocation of PKA-Cα in response to PTHrP treatment were also downregulated in the *Slc39a14*-KO cells (**Figure 4B**), although the PTH1R expression level was not diminished in these cells (**Figure 4B** **and Figure S2**).

PTHrP stimulation leads to elevated cAMP levels through adenylyl cyclase (AC) activation, and cAMP then activates PKA by binding to its regulatory subunit [41]. In the *Slc39a14*-KO chondrocytes, the basal cAMP level was significantly reduced (**Figure 4C** **, left**), and even after treatment with either PTHrP or the AC activator forskolin (FSK), the level was lower than in control chondrocytes, although the fold increase in cAMP levels after stimulation was comparable between the control and *Slc39a14*-KO chondrocytes (**Figure 4C** **, right**). These findings indicated that SLC39A14 was not required for the ligand-mediated AC activation, but rather, for maintaining the basal cAMP level in chondrocytes. Since PDE is known to control the cAMP level through its enzymatic activity [42], we next asked whether PDE activity was elevated in the *Slc39a14*-KO chondrocytes. As shown in **Figure 4D**, the *Slc39a14*-KO chondrocytes showed enhanced PDE activity and a reduced cAMP level compared with control cells, both of which were brought to normal levels by treatment with a PDE inhibitor, 3-isobutyl-1-methylxanthine (IBMX), suggesting that SLC39A14 negatively regulates PDE activity to upregulate the PTH1R-signaling pathway.

**Figure 5:**
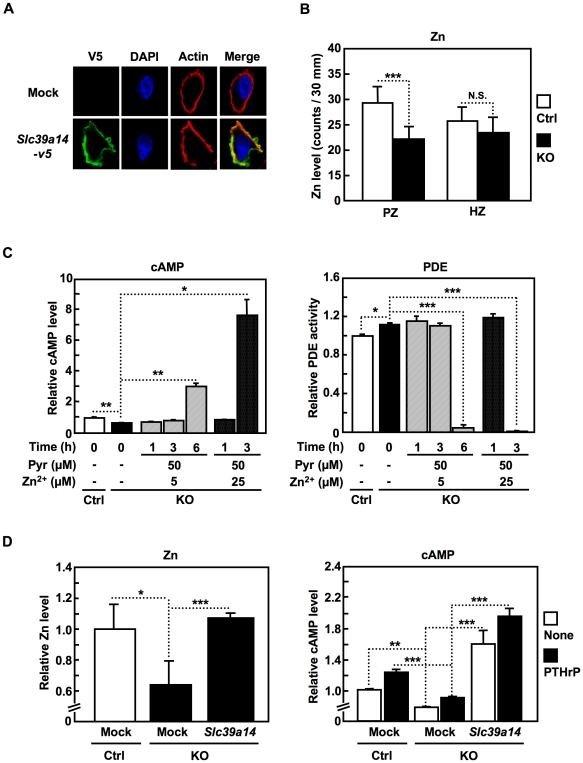
Zn positively regulates the cAMP level and facilitates PTH1R signaling. (**A**) Localization of SLC39A14 in transfected primary chondrocytes. SLC39A14 was labeled with anti-V5 (green), nuclei with DAPI (blue), and actin with phalloidin (red). (**B**) Electron probe X-ray microanalysis (EPMA) in the PZ and HZ of the growth plates from control (Ctrl) and *Slc39a14*-KO mice. The Zn levels in 10 cells in each zone were measured. Data represent the mean ± S.D. (***P<0.001). (**C**) Effect of Zn plus the ionophore pyrithione on the cAMP level and the PDE activity in control (Ctrl) and *Slc39a14*-KO chondrocytes. The cells were treated with Zn plus pyrithione at the indicated concentrations and time points. Data represent the mean ± S.D. (*P<0.05, **P<0.01, ***P<0.001). (**D**) Intracellular Zn and cAMP levels in control (Ctrl) and *Slc39a14*-KO chondrocytes after the transduction of empty (Mock) or *Slc39a14* cDNA-carrying lentivirus for 2 days. The intracellular Zn level was measured as the maximal emission at 516 nm (excitation at 494 nm) using a Varioskan after mixing FluoZin-3 with denatured cell lysates. The cAMP levels were measured after PTHrP treatment for 20 min. Data represent the mean ± S.D. (n = 3 per condition) (*P<0.05, **P<0.01, ***P<0.001).

SLC39A14 was localized to the plasma membrane (**Figure 5A**), and is thought to increase the intracellular Zn level by Zn influx [25], [26], raising the possibility that SLC39A14-mediated Zn influx plays a role in chondrocyte differentiation. To address this hypothesis, we measured the Zn level in control and *Slc39a14*-KO growth plates. Electron Probe X-ray Micro Analysis (EPMA) revealed that the intracellular Zn level was significantly decreased in the PZ of the *Slc39a14*-KO growth plate (**Figure 5B**), consistent with the idea that SLC39A14 functions to transport Zn from the outside to the inside of a cell. The forced introduction of Zn into cells using Zn plus pyrithione elevated the cAMP levels in *Slc39a14*-KO chondrocytes (**Figure 5C** **, left**) with a significant reduction of the PDE activity (**Figure 5C** **, right**) in a Zn-concentration- and time-dependent manner. Furthermore, the ectopic expression of SLC39A14 in *Slc39a14*-KO cells rescued the intracellular Zn (**Figure 5D** **, left**), and cAMP levels (**Figure 5D** **, right**).

The mRNA expression levels of other SLC39 members, which reside on the cell surface or in intracellular spaces, such as *Slc39a1, 6*, 7, *8*, *10*, and *13*, were not significantly altered in the *Slc39a14*-KO chondrocytes (**Figure S3**). Taken together, these results were consistent with the idea that SLC39A14 on the plasma membrane helps to maintain the steady-state level of PTH1R-mediated signaling by importing Zn to inhibit PDE activity, and strongly suggest that SLC39A14 plays a unique role in GPCR signaling in these cells, with little backup by the upregulation of other SLC39 family members to compensate for its loss of function.

### SLC39A14 positively regulates GH production via GHRHR signaling in the pituitary gland

**Figure 6:**
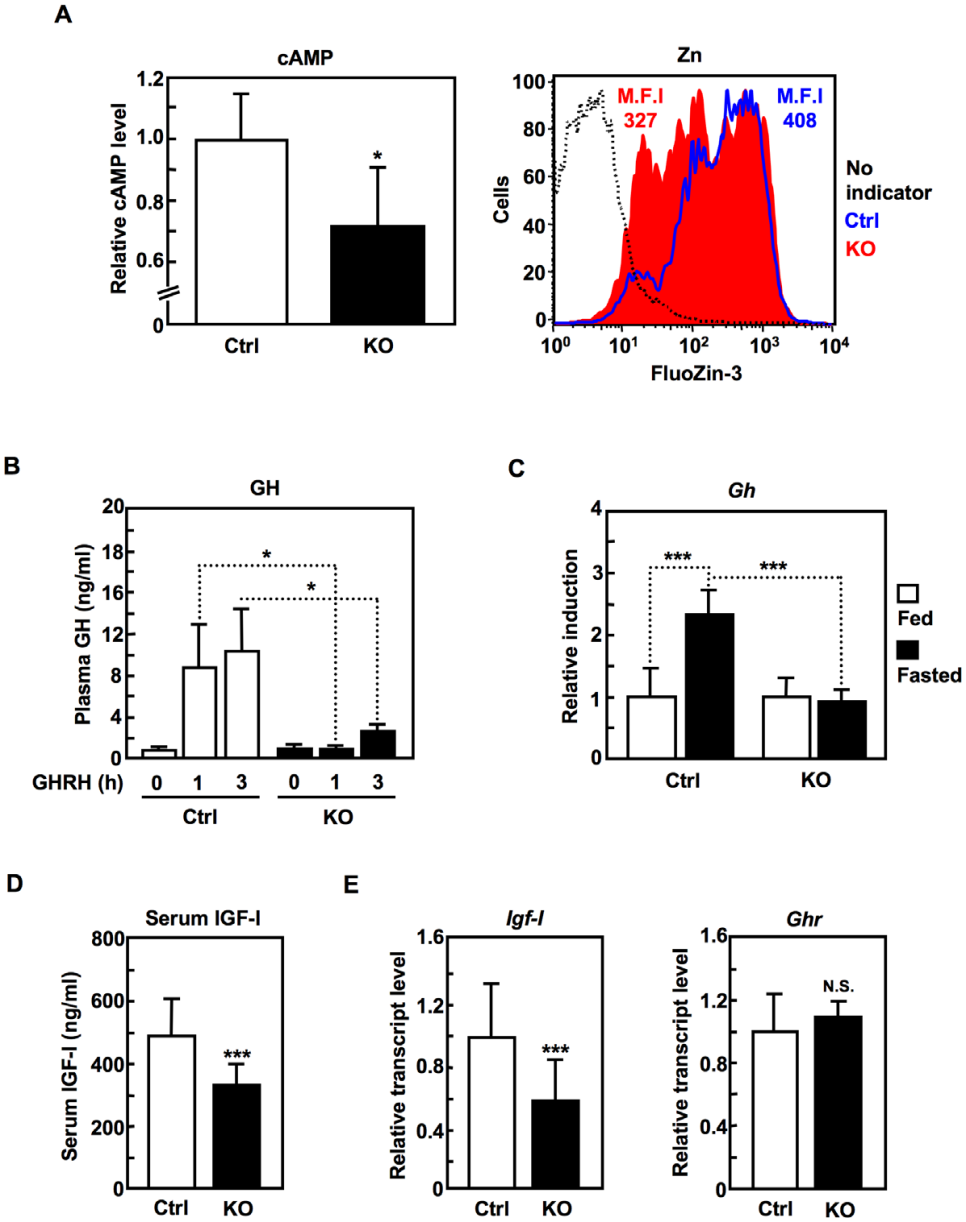
SLC39A14 positively regulates GH production via GHRHR signaling in the pituitary gland. (**A**) cAMP and intracellular Zn levels in pituitary cells (12-week-old control (Ctrl) and *Slc39a14*-KO mice (n = 3)). M.F.I. represents the mean fluorescent intensity. Data represent the mean ± S.D. (*P<0.05). (**B**) Human GHRH (500 µg/kg) was injected intravenously into 8-week-old control (Ctrl) and *Slc39a14*-KO mice (n = 8). Plasma was collected at the indicated times after injection, and the GH concentration was measured. Data represent the mean ± S.E.M. (*P<0.05). (**C**) *Gh* induction in the pituitary glands from 36-hours fasted control (Ctrl) and *Slc39a14*-KO mice (14–17-week-old, n = 3). Data represent the mean ± S.D. (***P<0.001). (**D**) Serum IGF-I concentration (3-week-old control (Ctrl), n = 9; *Slc39a14*-KO, n = 12). Data represent the mean ± S.D. (***P<0.001). (**E**) Hepatic *Igf-I* (n = 7) and *Ghr* (n = 3) levels in 3-week-old control (Ctrl) and *Slc39a14*-KO mice. Data represent the mean ± S.D. (***P<0.001).

Given that SLC39A14 negatively regulates PDE activity and that PDEs regulate several GPCR signaling pathways [42], we looked for likely GPCR pathways that might be affected by SLC39A14 expression. Since the *Slc39a14*-KO mice showed dwarfism, which is also seen in GH-deficient mouse models and humans [19], we investigated the role of SLC39A14 in signaling through growth hormone releasing hormone (GHRH) receptor (GHRHR), a GPCR expressed on pituitary somatotroph cells that induces the production and secretion of GH upon stimulation [27], [43]. We found that the levels of both pituitary cAMP and Zn were significantly lower in the *Slc39a14*-KO mice compared with control mice (**Figure 6A**). Furthermore, an intravenous bolus injection of GHRH elicited a smaller increase in the plasma GH level in the *Slc39a14*-KO mice than in control mice (**Figure 6B**). In addition, the induction of *Gh* during fasting, to which GHRH-mediated secretion contributes [44], was defective in the *Slc39a14*-KO mice (**Figure 6C**).

Notably, the *Slc39a14*-KO mice also showed a reduction in serum IGF-I (**Figure 6D**) and hepatic *Igf-I* expression levels (**Figure 6E**), which was not due to diminished *growth hormone receptor* (*Ghr*) expression, but could be explained by the impaired GH production (**Figure 6E**), because GH secretion stimulates IGF-I synthesis [45]. As seen in the chondrocytes, the mRNA expression levels of other SLC39 members were not significantly altered in the *Slc39a14*-KO pituitary cells (**Figure S3**). Collectively, these results indicated that SLC39A14 is selectively involved in the signaling through GHRHR, a GPCR in the pituitary gland.

### SLC39A14 positively regulates gluconeogenesis via GCGR signaling in the liver

**Figure 7:**
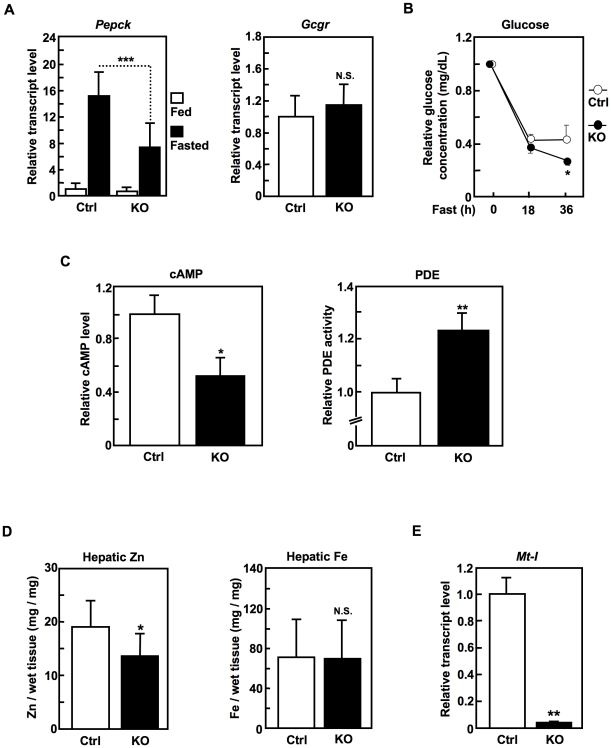
SLC39A14 positively regulates gluconeogenesis via GCGR signaling in the liver. (**A**) Hepatic *Pepck* and *Gcgr* levels in 18-hours fed or fasted control (Ctrl) and *Slc39a14*-KO mice (16-week-old, n = 2). Data represent the mean ± S.D. (***P<0.001). (**B**) Plasma glucose level in 18–36-hours fasted control (Ctrl) and *Slc39a14*-KO mice (7–52-week-old, n = 8). Data represent the mean ± S.E.M. (*P<0.05). (**C**) cAMP level and PDE activity in the liver from control (Ctrl) and *Slc39a14*-KO mice (4–8-week-old, n = 3). Data represent the mean ± S.D. (*P<0.05, **P<0.01). (**D**) Hepatic Zn and Fe levels in control (Ctrl) and *Slc39a14*-KO mice measured by ICP-MS. Data represent the mean ± S.D. (*P<0.05). (**E**) Hepatic *Mt-I* level in control (Ctrl) and *Slc39a14*-KO mice (16-week-old, n = 2). Data represent the mean ± S.D. (**P<0.01).

Since SLC39A14 is expressed in the liver (**Figure. 1B**) [25], [35], it may control gluconeogenesis, which occurs predominantly in the liver via signaling through the glucagon receptor (GCGR), a GPCR. Induction of the *phosphoenolpyruvate carboxykinase* (*Pepck*) gene in the liver is a critical event in the gluconeogenesis mediated by GCGR signaling [46]. As shown in **Figure 7A**, fasting-induced *Pepck* expression was significantly reduced in the *Slc39a14*-KO mice, although the *Gcgr* expression level did not change. In line with the lower *Pepck* induction, 36 hours of fasting significantly and continuously reduced the plasma glucose level in the *Slc39a14*-KO mice, whereas the plasma glucose level in the control mice plateaued after 18 hours (**Figure 7B**). These data indicated that fasting gluconeogenesis was significantly impaired in the *Slc39a14*-KO mice.

The *Slc39a14*-KO liver had a lower cAMP level, a higher PDE activity (**Figure 7C**), and a lower Zn level (**Figure 7D** **, left**) than the control liver. The reduced Zn level was confirmed by the reduction of the mRNA expression of *Mt-I*, a Zn indicator whose expression is induced by Zn via the metal response element (MRE) [47] (**Figure 7E**), whereas the level of iron (Fe) in the liver was not affected in the *Slc39a14*-KO mice (**Figure 7D**). Taken together, these data indicated that SLC39A14 positively controls gluconeogenesis through GCGR, and thus facilitates the hepatic cAMP-CREB signaling pathway.

## Discussion

**Figure 8:**
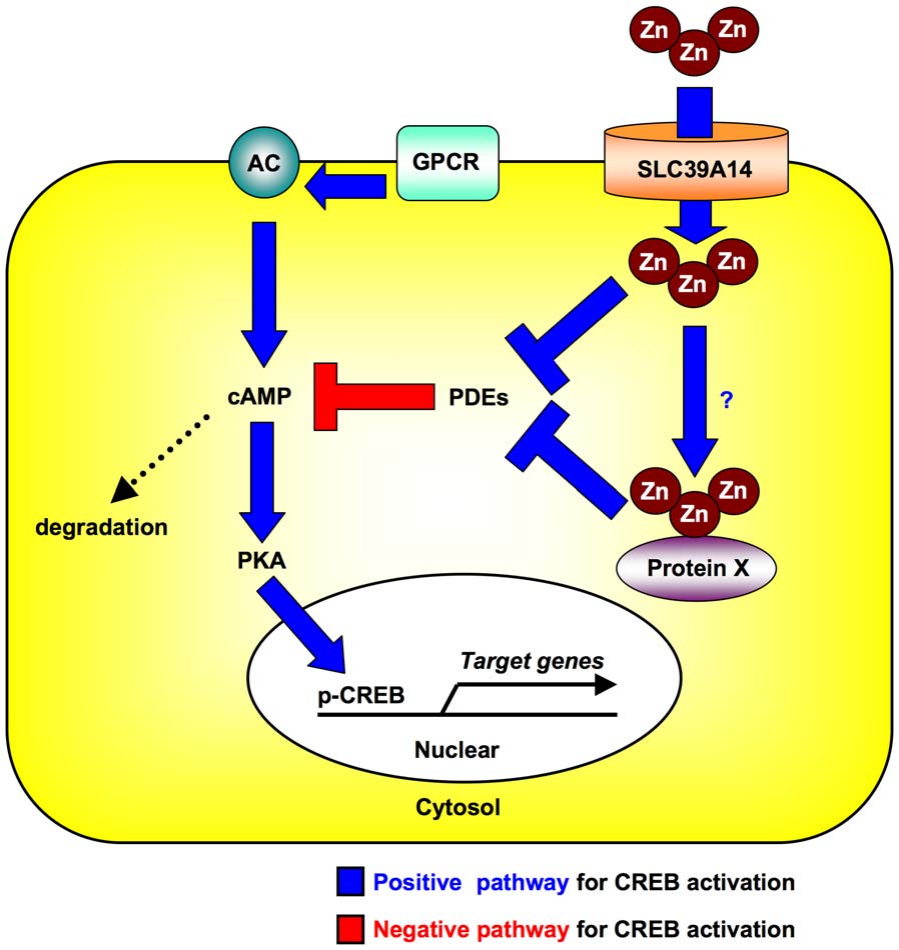
Schematic model for the regulation of GPCR-mediated signaling by SLC39A14. SLC39A14 regulates the basal cAMP level by suppressing PDE activity, through either the direct provision of Zn to PDE or the indirect provision via unidentified molecular chaperone(s) (Protein X). This system may facilitate the GPCR-cAMP-CREB pathway in endocrine-system reactions.

Our current study demonstrated that a Zn transporter, SLC39A14, controls GPCR-mediated signaling by maintaining the basal cAMP level and suppressing PDE activity. Our results showed that SLC39A14 is a novel endogenous regulator for systemic growth and energy homeostasis, and its role may provide a mechanism for the Zn-mediated regulation of endocrine signaling (**Figure 8**).

PTHrP-PTH1R signaling plays an important role in the endochondral ossification process, in which it blocks the premature hypertrophic differentiation of proliferative chondrocytes [37]. We concluded that SLC39A14 positively regulates PTHrP-PTH1R signaling based on the following findings. (1) The morphology of the *Slc39a14*-KO growth plate featured accelerated hypertrophy, characterized by the elongated pHZ and HZ, similar to observations in genetically manipulated-*Pth1r* mice [38], [39]. (2) The *Slc39a14*-KO growth plate showed increased expression levels of the hypertrophic markers *Ihh* and *Col10a1*. PTH1R signaling is reported to inhibit their expression levels [41], [48], consistent with the observation of elongated pHZ and HZ in the *Slc39a14*-KO mice. (3) *Slc39a14*-KO chondrocytes possessed a lower potential for PTH1R signal transduction.

The accelerated hypertrophy in *Pth1r* mutant mice might be caused not only by the accelerated differentiation of proliferative cells into hypertrophic cells, but also by the accelerated differentiation of resting cells into proliferative cells [39]. Given that Ihh acts to promote the differentiation of resting to proliferative cells and *Ihh*-KO mice display markedly reduced proliferation, with hypertrophy at inappropriate positions [49], it is reasonable to propose that the enhanced *Ihh* expression might stimulate the proliferation of resting chondrocytes accompanied by an increase in *Col2a1* expression (**Figure 3B**), leading to narrowing of the RZ and PZ (**Figure 3E**), in close coordination with accelerated hypertrophy in the *Slc39a14*-KO growth plate. However, the morphological abnormality of the *Slc39a14*-KO growth plate was less severe than that of the *Pth1r* mutant. It is possible that other Zn transporters and/or Zn-permeable channels [50] have similar functions (e.g. Zn transport activity) as the SLC39A14 protein, although their mRNA expression levels were not altered by the loss of SLC39A14 (**Figure S3**). This issue remains to be clarified. Nonetheless, the intracellular Zn level was significantly reduced in the PZ but not the HZ in the *Slc39a14*-KO mice (**Figure 5B**), reflecting the expression pattern of *Slc39a14* in the growth plate (**Figure 3B and 3C**). Our results collectively indicate that SLC39A14 plays an indispensable role in proper chondrocyte differentiation in the growth plate, by positively regulating PTH1R-mediated signaling.

Besides PTHrP-PTH1R signaling, the role of the GH-IGF-I axis in longitudinal bone growth is well established [18], [22]. It has been suggested that GH acts locally at the growth plate to induce IGF-I production, which then stimulates the proliferation of chondrocytes in a paracrine/autocrine manner, or induces resting chondrocytes to enter a proliferative state, independent of endocrine or paracrine IGF-I [22]. The Slc3914-KO mice showed significant decreases in their plasma concentrations of GH and IGF-I (aberrant GH-IGF-I axis), correlating with a low Zn level in the pituitary gland. In sharp contrast to mice lacking the *Ghr* gene, which have a normal birth weight and size [51], the *Slc39a14*-KO mice had a reduced birth weight and size (**Figure 2A** **and data not shown**). In addition, the growth plates of *Igf-I*-deficient mice display reduced hypertrophy [52], whereas hypertrophy was augmented in the *Slc39a14*-KO mice. Therefore, it is unlikely that the reduced GH and IGF-I levels impair chondrocyte differentiation in the *Slc39a14*-KO mice; rather, their role is probably related to the postnatal systemic growth retardation of these mice. However, we do not exclude the possibility that the reduced IGF-I level has an effect on growth during gestation, because *Igf-1*-deficient mice show intrauterine growth retardation with low birth weights [53]; therefore this issue requires further clarification. Nonetheless, it seems likely that in systemic growth, SLC39A14 plays an important role in controlling GH production by regulating the basal cAMP level in GHRHR-mediated signaling. This highlights SLC39A14′s importance as a positive GPCR regulator, not only in endochondral ossification, but also in GH production, thus concomitantly regulating systemic growth through these processes. Finally, our findings provide a mechanism that explains the reductions in GH and IGF-I in cases of Zn deficiency [20].

Here, we extended previous work on the importance of SLC39A14 in the signaling of a hepatic GPCR, GCGR, which controls gluconeogenesis during fasting. The liver regulates the metabolism of both Zn and Fe [54], [55]. We found that neither the hepatic (**Figure 7D**) nor the serum (**data not shown**) Fe level was altered in the *Slc39a14*-KO mice, suggesting that SLC39A14 specifically regulates the Zn metabolism in the liver at steady state. Overall, our results indicate that SLC39A14 may be a new player in the positive regulation of GPCR-mediated signaling in various systems (**Figure 8**).

It is noteworthy that the single ablation of the *Slc39a14* gene was sufficient to provoke abnormal chondrocyte differentiation. There are phenotypic similarities between the *Slc39a14*-KO mice and mice deficient in SLC39A13, another Zn transporter that is also required for mammalian growth. *Slc39a13*-KO mice show systemic growth retardation accompanied by impaired endochondral ossification [8]. In addition, *Slc39a14* and *Slc39a13* have similar distributions in the growth plate; they are both highly expressed in the PZ. However, the growth plate morphologies of the *Slc39a14*-KO mice are quite different from those of the *Slc39a13*-KO mice: the PZ shows narrowing in the *Slc39a14*-KO mice but elongation and disorganization in the *Slc39a13*-KO mice, and the HZ is elongated in the *Slc39a14*-KO mice, but is scanty in *Slc39a13*-KO mice, suggesting that SLC39A14 and SLC39A13 have distinct biological roles in growth control. These Zn transporters also have different cellular localizations. SLC39A14 is a cell-surface-localized transporter that controls the total cellular Zn content, whereas SLC39A13 localizes to the Golgi and regulates the local intracellular Zn distribution. Thus, the intracellular Zn status (e.g. accumulation and distribution of Zn) is controlled by various Zn transporters (e.g. SLC39A14 and SLC39A13), which influence distinct signaling pathways (GPCR by SLC39A14 and BMP/TGF-β by SLC39A13) leading to mammalian growth, in which many essential signaling events participate [18], [21], [22]. Furthermore, the expression level of *Slc39a13* was not changed in *Slc39a14*-KO cells (**Figure S3**), suggesting that SLC39A14 plays a unique biological role in controlling the GPCR signaling pathway, with little help from a backup system to compensate for its loss. The intracellular localization, expression level, Zn-transport activity, and post-translational modifications [4] may determine the specificity of each Zn transporter. Thus, our findings strongly suggest that SLC39A14 and SLC39A13 control skeletal growth by differentially regulating the Zn status to affect distinct signaling pathway(s), even though the growth phenotypes of their KO mice are similar. Our results support a new concept that different “Zn transporter-Zn status” axes act in unique signaling pathways to promote systemic growth.

In this study, it was not clarified how Zn acts through SLC39A14 to suppress PDE activity. SLC39A14 may regulate PDE activities by modulating the intracellular Zn level [56], [57] in tissues that express SLC39A14 and contain high concentrations of Zn [20], [58], [59]. As illustrated in **Figure 8**, the SLC39A14-mediated inhibitory effect may be due to the direct action of the transported Zn or to an indirect one via unidentified molecular chaperone(s) (Protein X) that receives Zn through SLC39A14 and provides it to PDE. Since GPCRs are expressed in numerous tissues, the *Slc39a14*-KO mice may be useful for studying GPCR-mediated biological events. Further studies on the mechanism by which SLC39A14 provides Zn to target molecules should help illuminate the regulation of GPCR-mediated signaling and Zn–associated biological events.

## Materials and Methods

### Mice

Mice were maintained on a 12-hour light-dark cycle, with a regular unrestricted diet available *ad libitum*. Mice at 6-8-weeks old were used for X-ray radiographic bone analysis, using a composite X-ray analyzing system (In vivo 3D Micro X-ray CT System R_mCT, Rigaku), and for bone histomorphometric analysis, using a semiautomatic image analyzing system (OsteoplanII; Carl Zeiss). Four-week-old mice were used for *in situ* hybridization analysis by the method of GENOSTAFF CO., LTD., and for analysis of the intracellular Zn level using an Electron Probe X-ray Micro Analyzer (JXA9200 II, JOEL). For analysis of the pituitary gland and liver, 3-52-week-old mice were evaluated under *ad libitum* or fasted conditions.

### Generation of *Slc39a14*-KO mice

The knockout mouse line of the *Slc39a14* gene was generated using previously described methods [60]. The RPCI-23 MM BAC (bacterial artificial chromosome) clone (Clone ID; 125B6), containing the mouse *Slc39a14* gene, was purchased from Invitrogen. A targeting vector was created to eliminate the genomic region encompassing exons 5–8 by inserting a *Neo*-cassette between exons 4 and 9 of *Slc39a14*, and deleting the intervening exons (**Figure. 1A**). This region contains the histidine-rich domain and conserved HEXPHEXGD motif that are common to SLC39 family members [36]. This vector was introduced into B6×129+Ter/SvJcl hybrid M1 ES cells, the cloned homologous recombinants were selected with antibiotics, and the genotypes were verified. We developed chimeric mice with the targeted ES-cell clones, and obtained homozygous mice by interbreeding the offspring. Heterozygous mice were phenotypically normal, and crosses between heterozygotes produced homozygous mutant mice according to Mendelian expectations. Genotyping was done by PCR using Taq polymerase (GE Healthcare). The primers used for genotyping were:

Forward: 5′- TGCTGCTGCTATTTGGGTCT -3′ for the wild-type allele

Forward: 5′- CTCGTGCTTTACGGTATCGC -3′ for the targeted allele and Reverse: 5′-GAATGCTGCATTGAAAAGGTC –3′, which was the same for the wild-type and targeted alleles.

### Ethics Statement

All animal experimental procedures were conducted according to guidelines approved by the RIKEN Institutional Animal Care and Use Committee (#22-013).

### Plasmid construction, transfection, and viral infection

The coding regions of the mouse *Slc39a14a* and *Slc39a14b* genes [35] were isolated from a liver cDNA library of the C57BL/6 mouse. To construct a C-terminally V5-tagged *Slc39a14a* (*Slc39a14a*–*v5*), the *Slc39a14a* fragments were amplified by PCR, followed by sequencing and cloning into the expression vector pcDNA6.2/V5-DEST (Invitrogen). To construct an N-terminally hemagglutinin (HA)-tagged *Slc39a14a* or *Slc39a14b* cDNA-lentiviral vector, the HA-tag sequence was inserted into the N-terminus of *Slc39a14a* or *Slc39a14b* by PCR, followed by ligating with CSII-CMV-IRES-hrGFP. Cells were transfected with expression vectors using Lipofectamine 2000 (Invitrogen). For lentiviral infections, 293T cells were transfected with *Slc39a14a* or *Slc39a14b* cDNA-containing CSII-CMV-IRES-hrGFP, pMDLg/p.RRE, pRSV-Rev, and pMD.G (VSV-G). After a 12-hour incubation, the culture medium was replaced with fresh medium, and then collected 24- and 48-hours later. The culture supernatants containing packaged lentivirus were pooled and concentrated using a Lenti-X concentrator (Clontech). The virus pellet was resuspended, and the virus titer was measured using the Lenti-X qRT-PCR Titration Kit (Clontech). Two or three days after starting the primary chondrocyte cell culture, the cells were infected with the *Slc39a14a* or *Slc39a14b* cDNA-carrying lentivirus in 8 µg/ml polybrene (Sigma) containing 10% FCS-α-MEM. After 2 days of infection, the cells were used for further analysis.

### Reagents and Antibodies

Human PTHrP (1-34) and (Nle^27^)-GHRH (1-29) amide were from Bachem. FSK and the broad-range PDE inhibitor (IBMX) were from Sigma. Pyrithione and Zn sulfate heptahydrate were from Molecular Probes and Wako Pure Chemical Industries, Ltd., respectively. Peptide N-glycosidase F (PNGase F) was from New England Biolabs. The anti-mouse SLC39A14 antibody was raised by immunizing rabbits with a KLH-conjugated peptide (CNSELDGKAPGTDEKV). Anti-CREB and anti-p-CREB (S133; 634-2) antibodies were from Millipore. The anti-PKA-Cα antibody was from Cell Signaling Technology. The anti-tubulin and anti-HDAC1 antibodies were from Sigma. The anti-PTH1R (K-20) and anti-V5 antibodies were from Abcam and Invitrogen, respectively.

### PNGase F treatment

Cell lysates were denatured in denaturing buffer (0.5% SDS and 40 mM DTT) at 37°C for 30 min. Nonidet P-40 and sodium phosphate (pH 7.5, 25°C) were added at final concentrations of 1% and 50 mM, respectively. Subsequently, PNGase F was added and the mixture was incubated at 37°C for 1 h. The samples were then denatured with SDS-PAGE loading buffer (37°C for 15 min) prior to immunoblotting.

### Measurement of cAMP level and PDE activity

The cAMP level and PDE activity in primary chondrocytes, pituitary cells, and liver were measured with the cAMP-Glo assay kit and PDE-Glo Phosphodiesterase assay kit (Promega), according to the manufacturer's instructions.

### RT-PCR and Quantitative RT-PCR (RT-qPCR) analyses

The total RNA was extracted from primary chondrocytes, pituitary cells, and liver using Sepasol-RNA I (Nacalai Tesque), and reverse-transcribed with an oligo-(dT) primer and reverse transcriptase (ReverTra Ace, Toyobo). RT-qPCR analysis was performed using the SYBR Green PCR Master Mix (Applied Biosystems). Samples were normalized to the *Gapdh* or *β-actin* expression. Primer sequences are available upon request.

### Enzyme-linked Immunosorbent assay (ELISA)

The serum IGF-I and plasma GH level were measured using the Mouse/Rat IGF-I Quantikine ELISA Kit (R&D Systems) and Rat/Mouse Growth Hormone ELISA kit (Millipore), according to the manufacturers' instructions.

### Measurement of Zn level

To measure the intracellular Zn level in pituitary cells, the pituitary gland was isolated and incubated with Trypsin/EDTA (Nacalai Tesque) at 37°C for 10 min. After being washed with PBS, the cells were loaded with 10 µM cell-permeant FluoZin^TM^-3 AM (Invitrogen) in plain RPMI medium (Sigma) at 37°C for 30 min. The cells were further incubated in fresh plain RPMI medium for 30 min prior to measuring the Zn level by flow cytometric analysis.

For the experiment using lentivirus-transduced chondrocytes, the cell lysates were boiled at 98°C for 10 min to inactivate the reporter protein, hrGFP, expressed from the IRES translation initiation sequence in the lentiviral vector. The cell lysates were diluted in cell-impermeant 10 µM FluoZin^TM^-3, tetrapotassium salt (Invitrogen) in 10 mM Tris-HCl (pH 7.4), and incubated for 30 sec prior to measuring the maximal emission at 516 nm (excitation at 494 nm) using a Varioskan (Thermo Electron Corporation). The hepatic Zn and Fe levels were measured by Inductively Coupled Plasma Mass Spectrometry (ICP-MS). An Electron Probe X-ray Micro Analyzer (JXA9200 II, JOEL) was used to measure the intracellular Zn level in the growth plate. Control- and *Slc39a14*-KO-derived growth plates were fixed with 1.25% glutaraldehyde and 1% paraformaldehyde in 0.1 M phosphate buffer, pH 7.4, then post-fixed in 2% osmium tetroxide, dehydrated in an ascending series of alcohol, and embedded in epoxy resin. The proliferative and hypertrophic chondrocytes in the embedded growth plate were scanned by an electron beam. The characteristic X-ray for Zn generated by the electron-beam exposure was detected.

### Histological and *in situ* hybridization analyses

Paraffin sections (4∼6 µm) processed from the tibia of 4-week-old mice or E17.5 embryos were prepared for hematoxylin and eosin (H&E) staining and for *in situ* hybridization analysis. Digoxigenin-labeled antisense RNA probes for *Slc39a14*, *Col2a1*, *Col10a1*, *Ihh* and *Pth1r* were used for *in situ* hybridization analysis by the method of GENOSTAFF CO., LTD. The sections used in the *in situ* hybridization analysis were counterstained with Kernechtrot stain solution. The probe sequences and hybridization conditions are available upon request.

### Isolation and culture of mouse primary chondrocytes, subcellular fractionation, immunoblotting, confocal microscopy, X-ray analysis, and bone histomorphometric analysis

Primary chondrocytes were isolated from the ribs as described previously [8]. The subcellular fractionation, immunoblotting, confocal microscopy, X-ray analysis, and bone histomorphometric analysis were performed using previously described methods [8].

### Statistical analysis

Differences among multiple groups were compared by 1-way ANOVA followed by a post-hoc comparison using Fisher's PLSD test. The two-tailed Student's t-test was used to analyze the difference between two groups.

## Supporting Information

Figure S1 **mRNA expression of** ***Slc39s*** **in pituitary cells and chondrocytes.** The mRNA expression of *Slc39s* in pituitary cells and chondrocytes was assessed by RT-PCR. The *Gh, Col2a1* and *Col10a1* mRNA levels are shown as controls for the pituitary cells and chondrocytes, respectively.
(TIF)

Figure S2 **Abnormal chondrocyte differentiation in the growth plate of** ***Slc39a14*** **-KO embryo.** *In situ* hybridization analysis for *Ihh* and *Pth1r* in the growth plates from E17.5 control (Ctrl) and *Slc39a14*-KO embryos.
(TIF)

Figure S3 **The mRNA expression levels of other Slc39s in Slc39a14-KO chondrocytes and pituitary cells.** (**A**) The mRNA expression levels of *Slc39s* in control (Ctrl) and *Slc39a14*-KO chondrocytes. Data represent the mean ± S.D. (N.S., no significance). (**B**) The mRNA expression levels of *Slc39s* in control (Ctrl) and *Slc39a14*-KO pituitary cells. Data represent the mean ± S.D. (N.S., no significance).
(TIF)
