# Supplementary figures and images for: The Zinc Transporter SLC39A14/ZIP14 Controls G-Protein Coupled Receptor-Mediated Signaling Required for Systemic Growth
Source: PLoS One. 2011 Mar 22;6(3):e18059. doi: 10.1371/journal.pone.0018059 (PMC3062567; doi:10.1371/journal.pone.0018059)

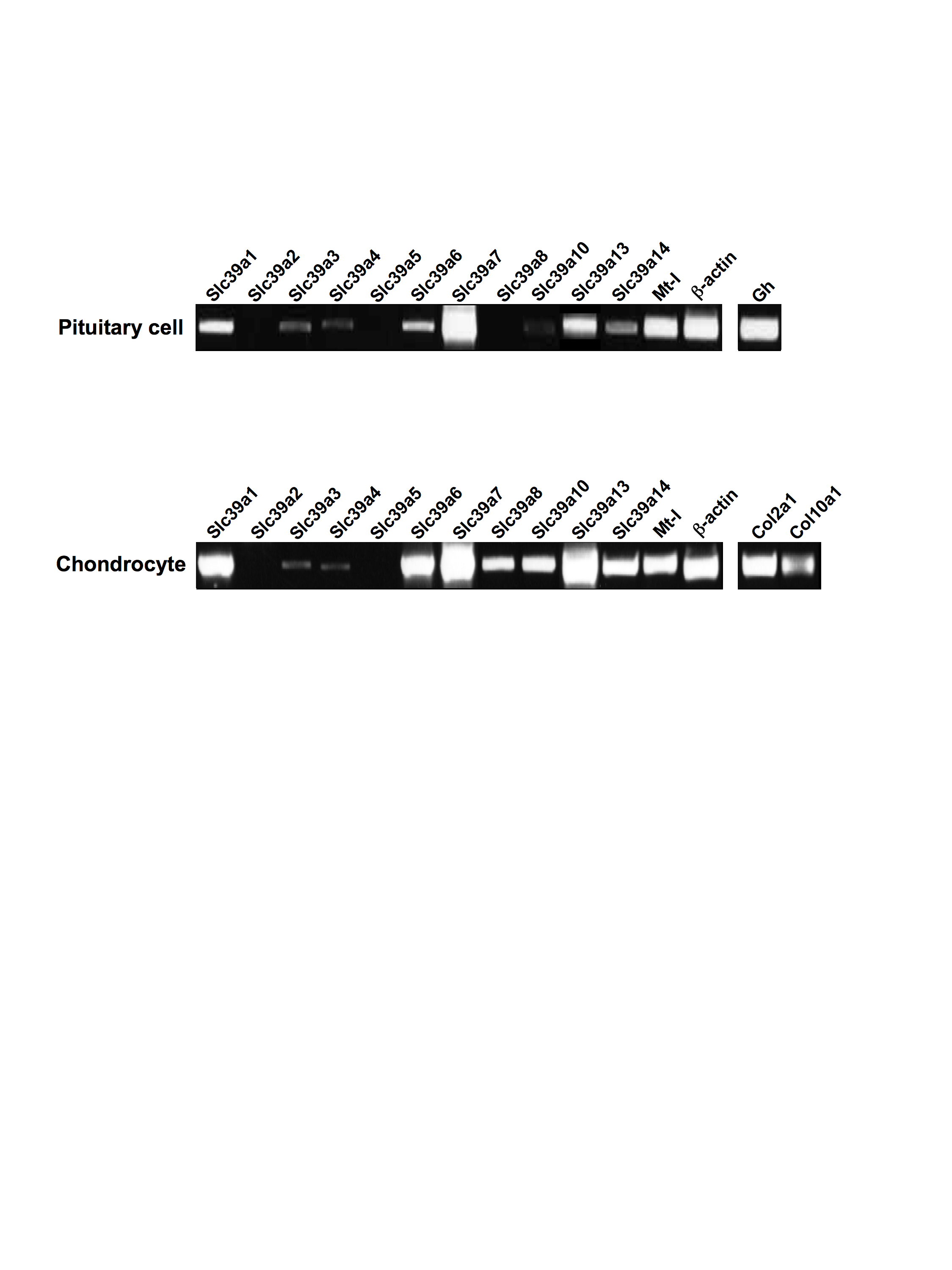

Supplement: Figure S1 — mRNA expression of Slc39s in pituitary cells and chondrocytes. The mRNA expression of Slc39s in pituitary cells and chondrocytes was assessed by RT-PCR. The Gh, Col2a1 and Col10a1 mRNA levels are shown as controls for the pituitary cells and chondrocytes, respectively. (TIF) [file pone.0018059.s001.tif]

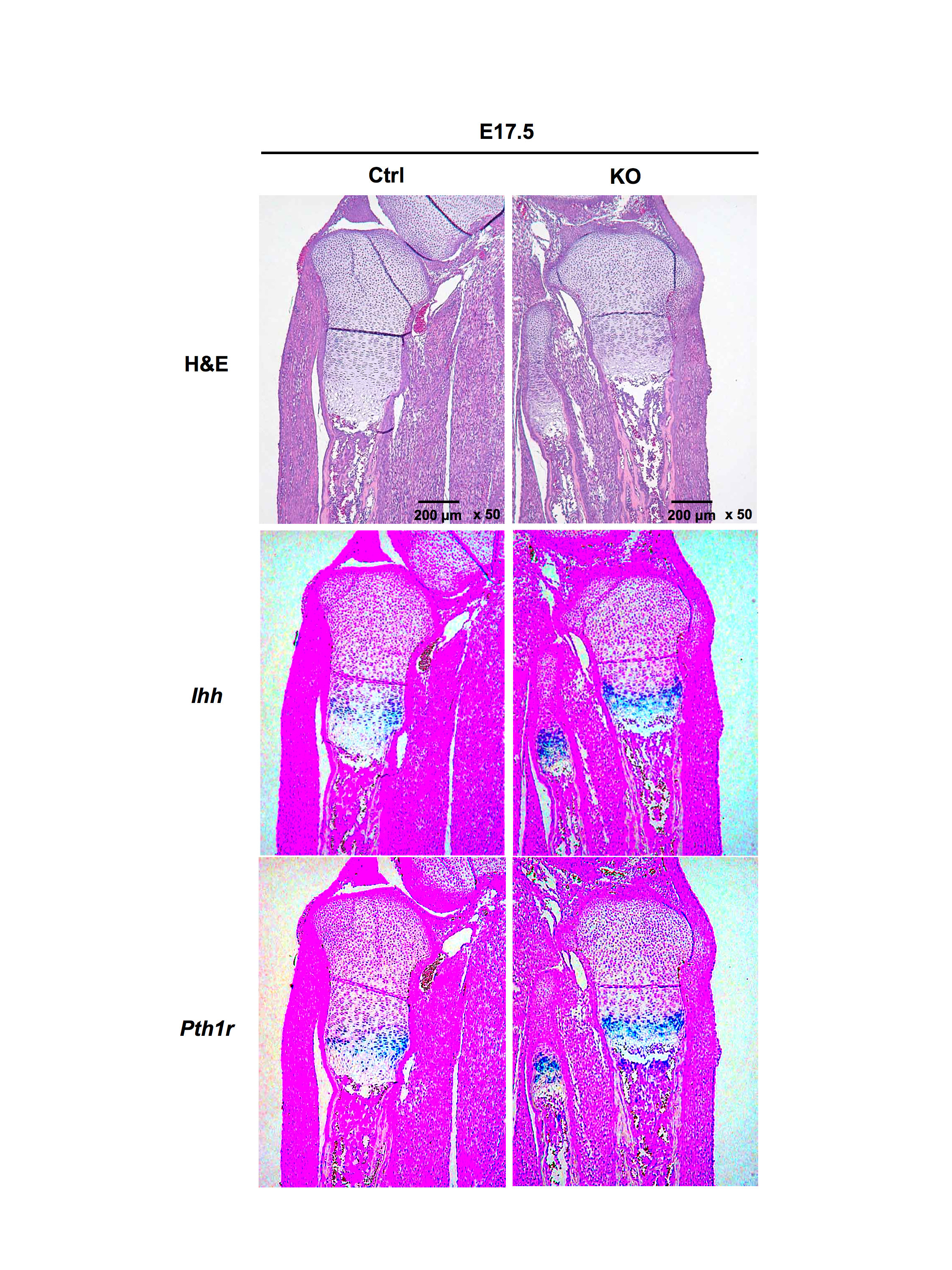

Supplement: Figure S2 — Abnormal chondrocyte differentiation in the growth plate of Slc39a14 -KO embryo. In situ hybridization analysis for Ihh and Pth1r in the growth plates from E17.5 control (Ctrl) and Slc39a14-KO embryos. (TIF) [file pone.0018059.s002.tif]

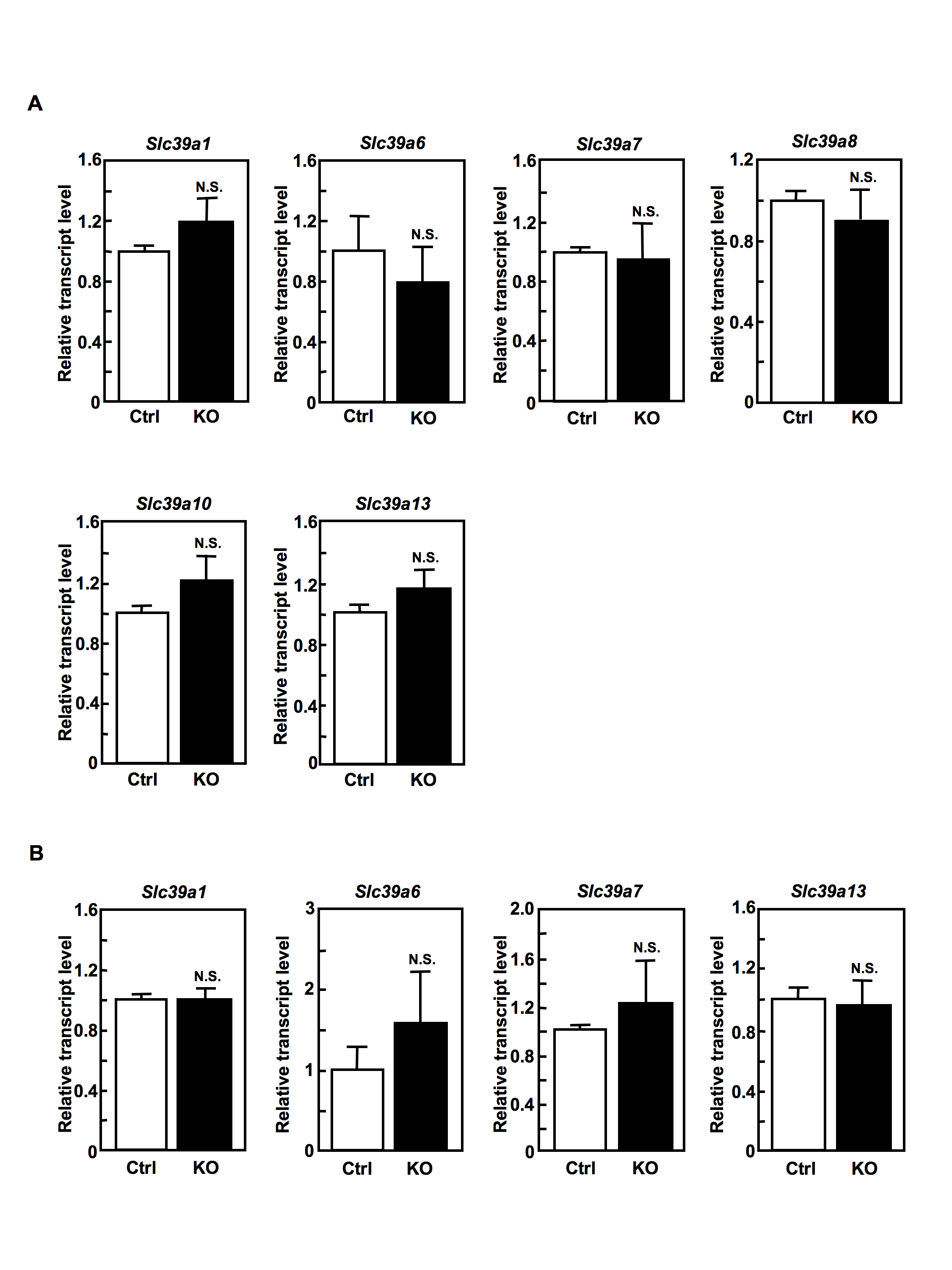

Supplement: Figure S3 — The mRNA expression levels of other Slc39s in Slc39a14-KO chondrocytes and pituitary cells. (A) The mRNA expression levels of Slc39s in control (Ctrl) and Slc39a14-KO chondrocytes. Data represent the mean ± S.D. (N.S., no significance). (B) The mRNA expression levels of Slc39s in control (Ctrl) and Slc39a14-KO pituitary cells. Data represent the mean ± S.D. (N.S., no significance). (TIF) [file pone.0018059.s003.tif]
